# Ion Exchange Chromatography and Mass Spectrometric Methods for Analysis of Cadmium-Phytochelatin (II) Complexes

**DOI:** 10.3390/ijerph10041304

**Published:** 2013-03-28

**Authors:** Miguel Angel Merlos Rodrigo, Natalia Cernei, Marketa Kominkova, Ondrej Zitka, Miroslava Beklova, Josef Zehnalek, Rene Kizek, Vojtech Adam

**Affiliations:** 1 Department of Chemistry and Biochemistry, Faculty of Agronomy, Mendel University in Brno, Zemedelska 1, CZ-613 00 Brno, Czech Republic; E-Mails: miguelangel.merlos@eez.csic.es (M.A.M.R.); zitkao@seznam.cz (O.Z.); zehnalek@mendelu.cz (J.Z.); kizek@sci.muni.cz (R.K.); 2 Central European Institute of Technology, Brno University of Technology, Technicka 3058/10, CZ-616 00 Brno, Czech Republic; E-Mails: cernei.natalia3@gmail.com (N.C.); beklovam@vfu.cz (M.B.); 3 Department of Veterinary Ecology and Environmental Protection, Faculty of Veterinary Hygiene and Ecology, University of Veterinary and Pharmaceutical Sciences, Palackeho 1-3, CZ-612 42 Brno, Czech Republic; E-Mail: kominkova.marketa@gmail.com; 4 Lead and Cadmium Initiatives, United Nations Environment Program, Faculty of Agronomy, Mendel University in Brno, Zemedelska 1, CZ-613 00 Brno, Czech Republic

**Keywords:** ion exchange chromatography, mass spectrometry, MALDI-TOF, phytochelatin, cadmium, intramolecular complex

## Abstract

In this study, *in vitro* formed Cd-phytochelatin (PC2) complexes were characterized using ion exchange chromatography (IEC) and matrix-assisted laser desorption/ionization time-of-flight (MALDI-TOF) mass spectrometry. The ratio of both studied compounds as well as experimental conditions were optimized. The highest yield of the complex was observed under an applied concentration of 100 µg·mL^−1^ PC2 and 100 µg·mL^−1^ of CdCl_2_. The data obtained show that IEC in combination with MALDI-TOF is a reliable and fast method for the determination of these complexes.

## 1. Introduction

Cadmium still presents a threat to the environment. This metal can be accumulated by plants, resulting in damage to important cellular biochemical pathways. Therefore, plants, algae and fungi use intracellular metal-binding peptides, known as phytochelatins (PCs), for maintaining metal homeostasis and for detoxification of the accumulated cadmium(II) ions [1]. PCs are enzymatically synthesized peptides produced rich in the -SH group and having the structure of (γ-Glu-Cys)n-Gly, where n varies from two to five [1,2,3]. The role of PCs in metal detoxification results from immobilization of metals and, thus, preventing non-specific binding to important biomolecules, followed by the transport of the metal-PC complexes into the vacuole, or its excretion [4,5,6,7,8,9,10]. It is therefore of interest to attempt to characterize Cd-PC complexes.

Several analytical methods have been used to analyze PC and complexes of metal [11,12,13,14,15,16]. In *Nicotiana tabacum* grown under different doses of cadmium(II) ions, PC2 content was determined by using high performance liquid chromatography (HPLC) with electrochemical detection [17]. Other authors used a mass spectrometer coupled with HPLC to study GSH complexes [18] or stationary electrochemistry voltammetric analysis with subsequently ESI-MS for confirmation of PC2 with Cu^2+^, Cd^2+^ and Pb^2+^ complexes [19]. An approach based on size-exclusion chromatography with off-line detection of phytochelatins, by reverse phase HPLC, and metal ions, by atomic absorption spectrometry, was used for studying the formation of Cd and PC complexes [20]. Size exclusion chromatography of the tissue extracts from seedlings exposed to 100 µM Cd revealed the presence of Cd-PC complexes in flax cultivars [21]. Raab *et al.* developed a method for separation (PCs)-metal(loid) complexes by HPLC system using parallel metal(loid)-specific (inductively coupled plasma-mass spectrometry) and organic-specific (electrospray ionization-mass spectrometry) detection approaches and used it to identify the nature of arsenic (As)-PC complexes in plant extracts [22]. Ion-exchange chromatography (IEC) coupled with colorimetric detection using post-column derivatization with ninhydrin is usually used for determination of free amino acids and other amino acids like compounds in biological samples [23]. IEC is a fast and effective method for PC and PC metal-complexes determination [24].

In this study, Cd-PC2 complexes synthesized *in vitro* were analyzed using IEC and mass spectrometry using a matrix-assisted laser desorption/ionization time-of-flight (MALDI-TOF) mass spectrometer. The ratio of both studied compounds as well as the experimental conditions were optimized.

## 2. Experimental Section

### 2.1. Chemicals

Phytochelatin2 (γ-Glu-Cys)_2_ (PC2) was synthesized in Clonestar (Brno, Czech Republic) with a purity higher than 90 %. CdCl_2_ and other chemicals used in this study were ACS grade and purchased from Sigma Aldrich (St. Louis, MA, USA) unless noted otherwise. Standard solutions of PC2 and CdCl_2_ were prepared daily by dilution of the stock solutions with ACS water. All buffers were prepared in ultrapure water (Mili-Q) obtained using reverse osmosis equipment Aqual 25 (Aqua Osmotic, Tišnov, Czech Republic) with further purification by using apparatus MiliQ Direct QUV equipped with the UV lamp (Millipore Corp., Billerica, USA). The resistance was 18 MΩ. The pH was measured using a WTW inoLab (Weilheim, Germany) pH meter. For preparation of the complex of PC2 with CdCl_2 _we used the following chemicals: PC2 (300 µg·mL^−1^), CdCl_2_ (300 µg·mL^−1^) and serine (300 µg·mL^−1^) (internal standard was added before analysis only for determination using IEC). Concentration of the working solution of CdCl_2_ was used within the range from 3 to 100 µg·mL^−1^. Complexes were mixed using vortex BioVortex V1 (Biosan, Riga, Latvia) for 1 min, further, the complexes were interacted for 1 h at room temperature.

### 2.2. Ion Exchange Chromatography

For determination of PC2 an ion-exchange liquid chromatograph (Model AAA-400, Ingos, Prague, Czech Republic) with post column derivatisation with ninhydrin and VIS detector was used. A glass column with inner diameter of 3.7 mm and 350 mm in length was filled manually with a strong cation exchange resin in sodium cycle LG ANB (Ingos) with approximately 12 µm particles and 8% porosity. The column was tempered within the range 40–70 °C. A double channel VIS detector with 5 µL cell was set to detection wavelengths 440 nm and 570 nm. A solution of ninhydrin (Ingos) was prepared with 75 % (*v*/*v*) methylcelosolve (Ingos) and with 2% (*v*/*v*) 4 M acetic buffer (pH 5.5). Tin chloride (SnCl_2_) was used as a reducing agent. The prepared solution of ninhydrin was stored under an inert atmosphere (N_2_) in dark at 4 °C. The eluting mobile phase was containing 11.11 g of citric acid, 4.04 g of sodium citrate, 9.25 g of NaCl, 0.1 g of sodium azide, 2.5 ml of thiodiglycol per liter of solution and pH was 2.7. The flow rate of mobile phase was 0.25 mL·min^−1^ and flow rate of ninhydrin was tested within the range from 0.1 to 0.35 mL·min^−1^. The reactor temperature was optimized within the range from 90 to 130 °C. The volume of injection of the sample was 100 µL.

### 2.3. Matrix-Assisted Laser Desorption/Ionization Time-of-Flight Mass Spectrometry

The mass spectrometry experiments were performed on a MALDI-TOF/TOF mass spectrometer Bruker Ultraflextreme (Bruker GmbH, Bremen, Germany) equipped with a laser (Bruker GmbH, Bremen, Germany) operating at wavelength of 355 nm with an accelerating voltage of 25 kV, cooled with nitrogen and a maximum energy of 43.2 µJ with repetition rate 2,000 Hz in linear and positive mode, and with software for data acquisition and processing of mass spectra flexControl version 3.4 and flexAnalysis version 2.2. The matrix used in the MALDI method was α-cyano-4-hydroxycinnamic acid (CCA) supplied by Bruker. The matrix was prepared in 70% methanol. Mixture was thoroughly vortexed and ultrasonicated using Bandelin 152 Sonorex Digital 10P ultrasonic bath (Bandelin, Berlin Germany) for 2 min at room temperature. The samples of the complexes were prepared with TA30 (30% acetonitrile, 0.1% trifluoroacetic acid solution). The solutions for analysis were mixed in ratio of 1:1 (matrix/substance). After obtaining a homogeneous solution, 1 µL was applied on the target and dried under atmospheric pressure and room temperature. A mixture of peptide calibrations standard (Bruker) was used for calibration the instrument. The MS spectra were typically acquired by averaging 20 sub spectra from a total of 500 shots of the laser (Smartbeam 2, Version: 1_0_38.5).

## 3. Results and Discussion

### 3.1. Optimization of IEC for Cd-PC Complex Determination

**Figure 1:**
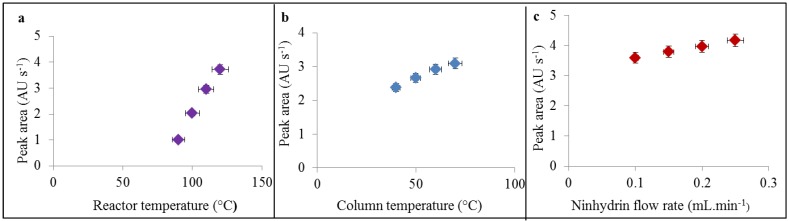
(**a**) Influence of reactor temperature, (**b**) column temperature and (**c**) ninhydrin flow rate on height of PC2 (50 µg·mL^−1^) peak. The peptide was measured by IEC.

Post-column derivatization used in IEC systems is based on the reaction of ninhydrin (2,2-dihydroxy-1,3-indandione) with nitrogen from an amino acid. Nitrogen from the amino moiety is oxidized under slightly acidic conditions maintained by acetate buffer (pH 4.3), under reduction conditions (maintained by SnCl_2_), and higher temperatures maintained by flow reactor (90–130 °C) resulting in the stable product called as Ruhemann’s red, which is well detectable in visible spectra under 570 nm. It principally works for all amines (peptides, biogenic amines even for ammonium ions). Therefore we used this method for detection of PC2 peptide in our IEC study. In our previously published paper [25], we found that the reactor temperature, column temperature and ninhydrin flow ratio belong to the most analysis influencing factors. In comparison to our previous paper we found the different effects of these factors on the sensitivity of detection, which are caused by various analytes. In the case of reactor temperature, which was tested within the range from 90 to 130 °C, we found that maximum peak area was increasing due to applied temperature almost linearly up to the limit of reactor as 130 °C (Figure 1(a)). This trend was different from this one we found in the study with taurine, where the maximum was found to be 110 °C [25], and then yield of derivatisation reaction was almost stable up to the 130 °C. This difference might by caused by higher efficiency of disintegration of peptide bonds, which are not so willing to react with ninhydrin as free amino groups under lower temperatures. Temperature of column, which was tested ranging from 40 to 70 °C, influences the detection based on the temperature initiation before post column mixing. It is obvious that it positively affected the detection with linear increasing trend (Figure 1(b)).

**Figure 2:**
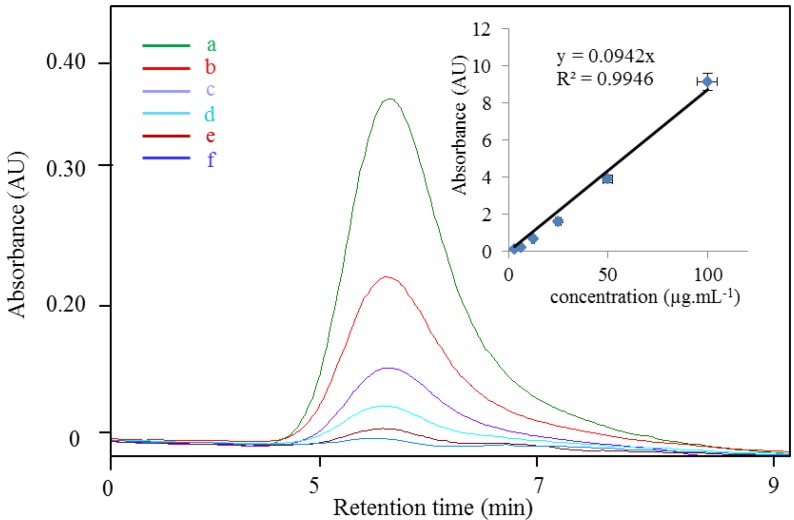
Calibration curve of PC2 in IEC: (a) 100, (b) 50, (c) 25, (d) 12.25, (e) 6 and (f) 3 µg·mL^−1^ PC2 in H2O. The experimental conditions were as follows: reactor temperature 130 °C, column temperature 70 °C, ninhydrin flow rate 0.3 mL·min^−1^.

Ninhydrin flow as the last tested parameter exhibits the slightly linear increasing trend in detection efficacy within the tested range from 0.1 to 0.3 mL·min^−1^ with the upper mentioned value as the maximum (Figure 1(c)). The optimal conditions (reactor temperature 130 °C, column temperature 70 °C, ninhydrin flow rate 0.3 mL·min^−1^) were used for analysis of Cd-PC complexes. Primarily, we determined calibration curve of the PC2 under the optimal conditions. Calibration curve was determined as the dependence of the peak area on the concentration of PC2 (inset in Figure 2) andexhibited good linearity (R^2^ = 0.9946) and R.S.D 1.8% (n = 6). Retention time of determined PC2 was 6.22 ± 0.30 min (Figure 2).

### 3.2. Study of Cd-PC2 Complexes by IEC and MALDI-TOF

**Figure 3:**
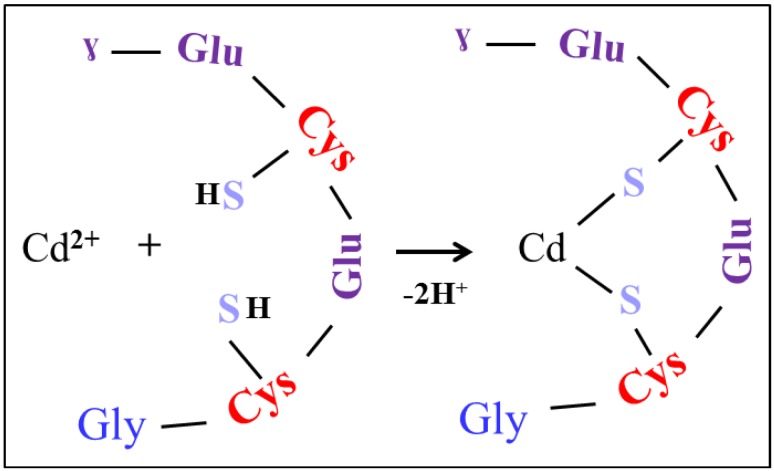
Model of complex between cadmium Cd(II) ion and one molecules of PC2. Cys (cysteine), Glu (glutamic acid), Gly (glycine) and S (sulfur).

**Figure 4:**
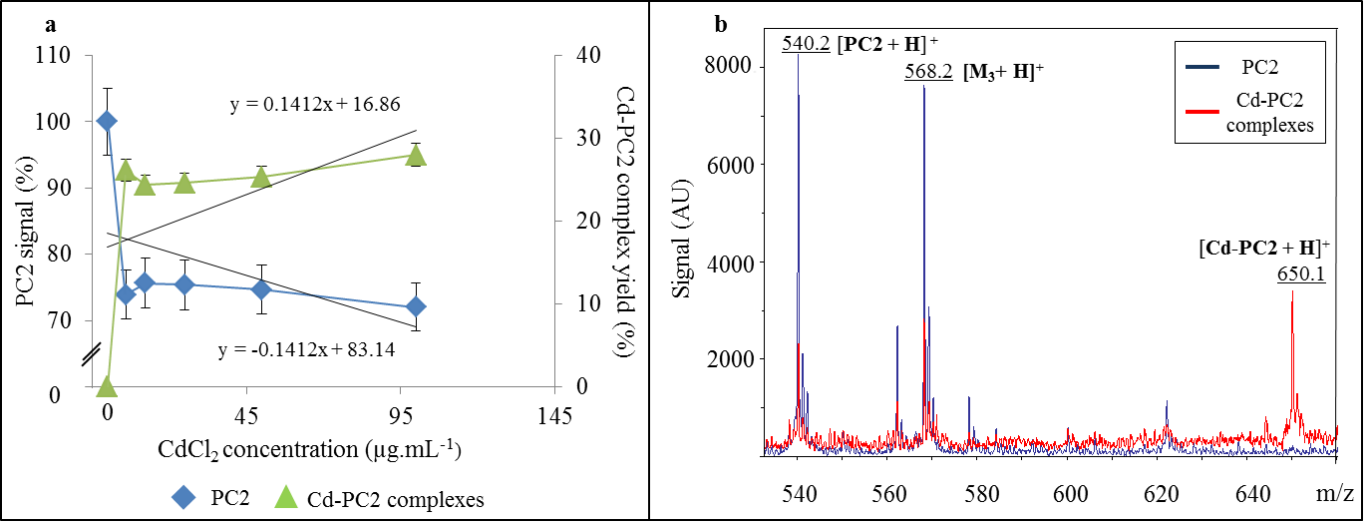
(**a**) Diagram of change in the signal of PC2 (%) and yield of Cd-PC2 complex (%) as function of CdCl_2_ concentrations determined by IEC. (**b**) Mass spectra of PC2 (blue line) and Cd-PC2 complexes (red line) in CCA matrix measured by MALDI-TOF.

To understand the nature of the possible Cd-PC complexes in Cd-stressed biological samples it is important to determine the formation of these complexes *in vitro*. Our hypothesis of the formation of Cd-PC2 is the binding of Cd to sulfhydryl groups of the two cysteine molecules that are a part of the PC2 with the loss of two hydrogen protons. Thus, we suggested that an intermolecular complex can be formed only as it is shown in Figure 3. Therefore we prepared and analyzed different complex mixtures with constant concentration of PC2 100 µg·mL^−1^ with addition of Cd^2+^ (in the form of CdCl_2_) in concentration of 0, 5, 10, 25, 50 and 100 µg·mL^−1^ using of optimized IEC method. We observed none effect on the retention tine of the peak of PC2. Surprisingly we observed the nonlinear decreasing of the peak area. The decrease of the signal of PC2 in the presence of CdCl_2_ was probably due to complexes formation accompanied with the increasing yield of suggested complex as it is shown in Figure 4(a). This was probably caused by inhibition of derivatisation reaction of peptide, which has been complexed with Cd(II) ions. No decreasing effect was observed on peak of internal standard of serine. The signal of PC2 decreased with the increasing concentration of CdCl_2_ in the solution, showing the highest decrease in 100 µg·mL^−1^ CdCl_2_ (28%). It is evident that CdCl_2_ induced Cd-PC2 complexes formation. The increase of yield of Cd-PC2 complexes were suggested as a function of the CdCl_2_ content of the solution, being highest in concentration 100 µg·mL^−1^ (97%).

To verify that Cd-PC2 complexes were formed, we analyzed it by MALDI-TOF. The main observed signals shown in Figure 4(b) were assigned as follows: [PC2 + H]^+^ (*m/z* 540.2), [M_3_ + H]^+^ (*m/z* 568.2) (correspond to matrix cluster trimer) and [Cd-PC2 + H]^+^ (*m/z* 650.1). It is evident that a suggested intramolecular complex, Cd-PC2, was found, as it shown in Figure 4(b) (red line). The presence of observed mass of 650.1 Da confirms the abundance of intramolecular complexes according to scheme shown in Figure 3.

## 4. Conclusions

The optimum conditions for detection of intermolecular Cd-PC2 complexes prepared *in vitro* were determined for ion exchange chromatography as well as for matrix-assisted laser desorption/ionization-time of flight mass spectroscopy These two methods have been demonstrated to be ideal and promising techniques for screening and characterizing *in vitro* peptide-metal complexes. It was demonstrated that both techniques had the ability to identify the formation of Cd-PC2 complex.
